# Supplementary material for: Structure–function insights reveal the human ribosome as a cancer target for antibiotics
Source: Nat Commun. 2016 Sep 26;7:12856. doi: 10.1038/ncomms12856 (PMC5052680; doi:10.1038/ncomms12856)
Supplement: Supplementary Figures — 1-8 [file ncomms12856-s1.pdf]

Supplementary Information

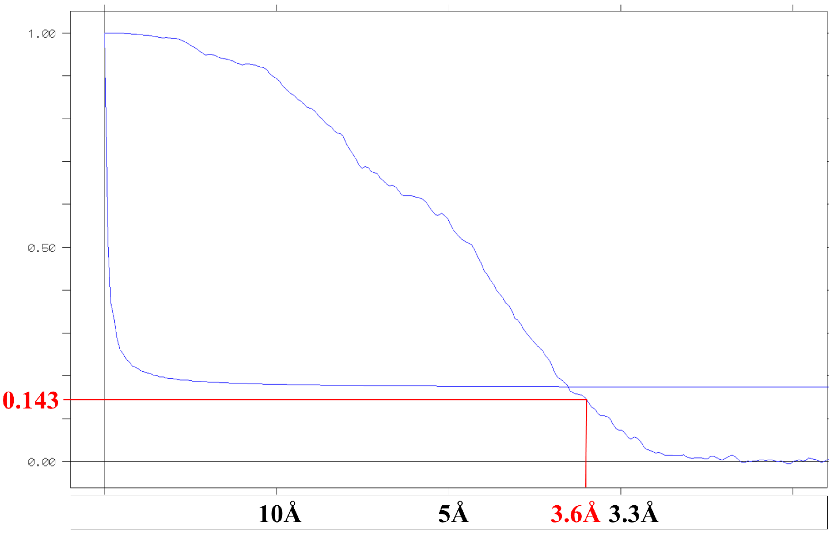

Resolution estimation

The average resolution of the cryo-EM map as estimated from Fourier shell correlation according to the 0.143 (in red) and half-bit criteria (see methods).

Supplementary Fig. 1

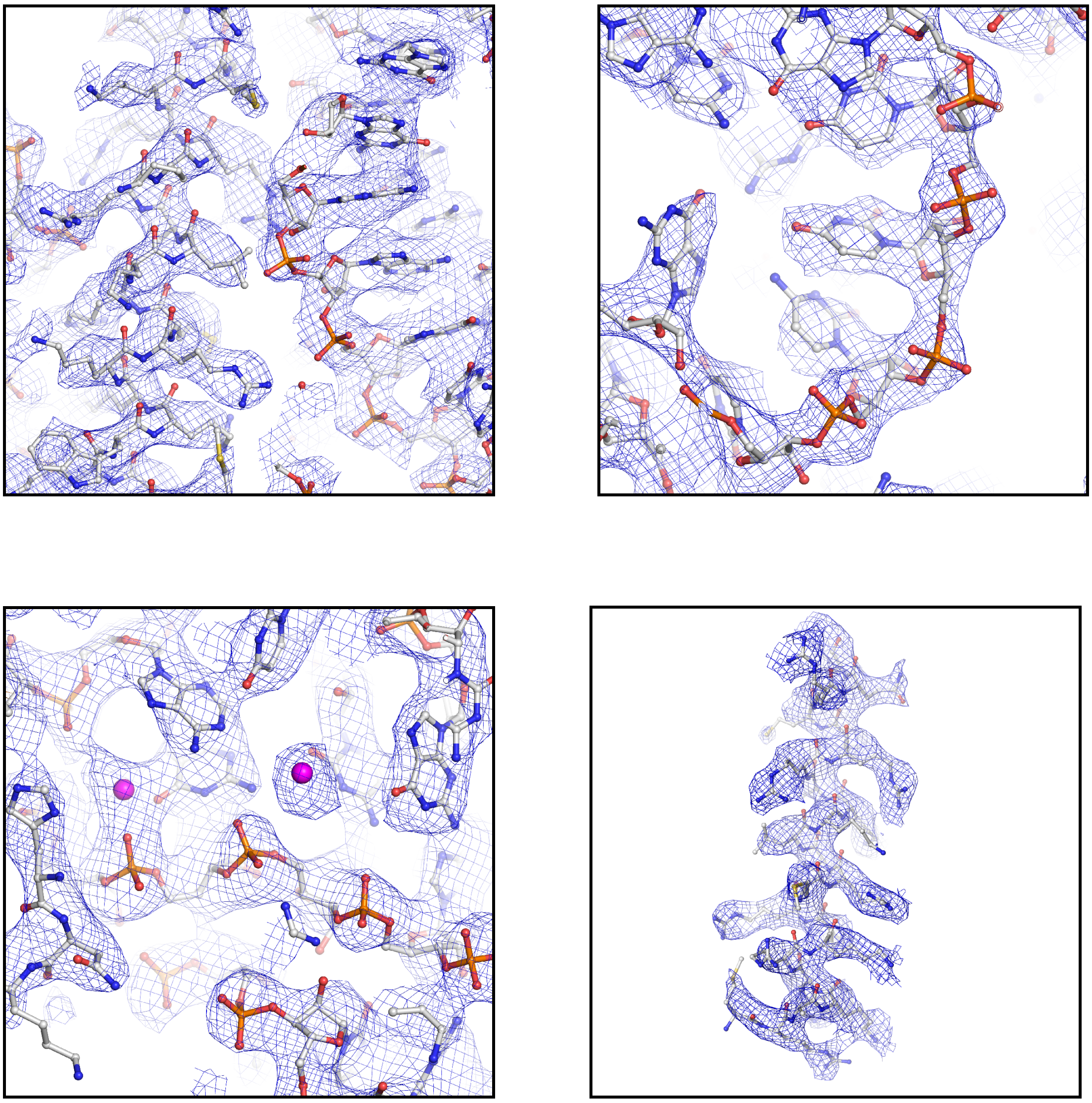

Typical features in the cryo-EM map of the 80S/CHX complex

Some typical regions are shown (top two panels: protein eL5 region and adjacent rRNA; bottom left: protein S2 and rRNA, bottom right: protein eL41). Most amino acid and nucleic acid side chains are resolved, and Mg^{2+} ions can be localized.

Supplementary Fig. 2

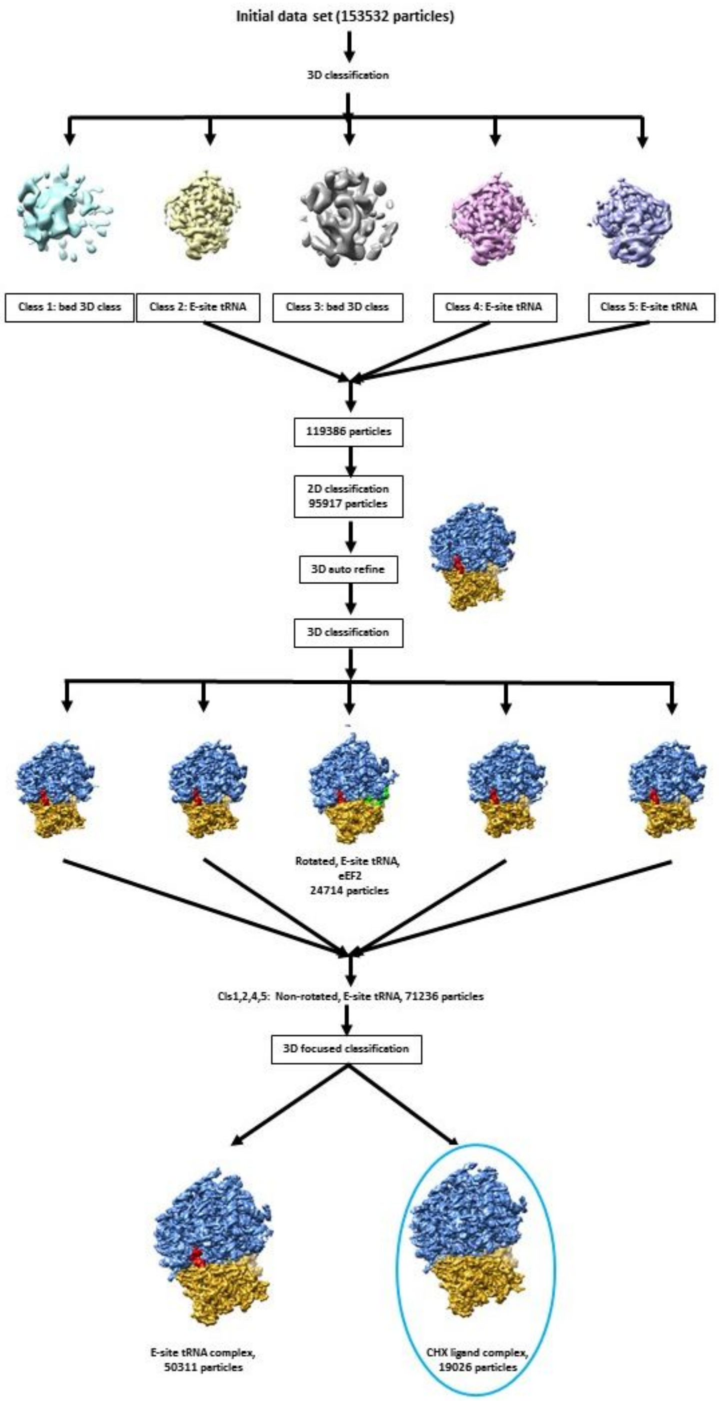

Particle sorting scheme

Particle sorting started from ~153 000 particles, finally resulting in two non-rotated ribosome 3D classes with ~50 000 and ~19 000 particles containing either E-site tRNA (red) or the CHX ligand refined to high resolution (see methods).

Supplementary Fig. 3

KO99L tPTEN-/-					
IC50 (nM)	CHX	DON	HHT	VA	ANS
Mitochondrial metabolism	248	479	15.7	23.9	33.2
cell death	1734	305.7	0.41	22.5	59.8
cell proliferation	20.7	78.8	17.5	25.3	52.2

IC50 values of different antibiotics

IC_{50} values for the different E-specific antibiotics on tPTEN-/- KO99L cells corresponding to the experiments displayed in the main **Figs. 3A-C**.

Supplementary Fig. 4

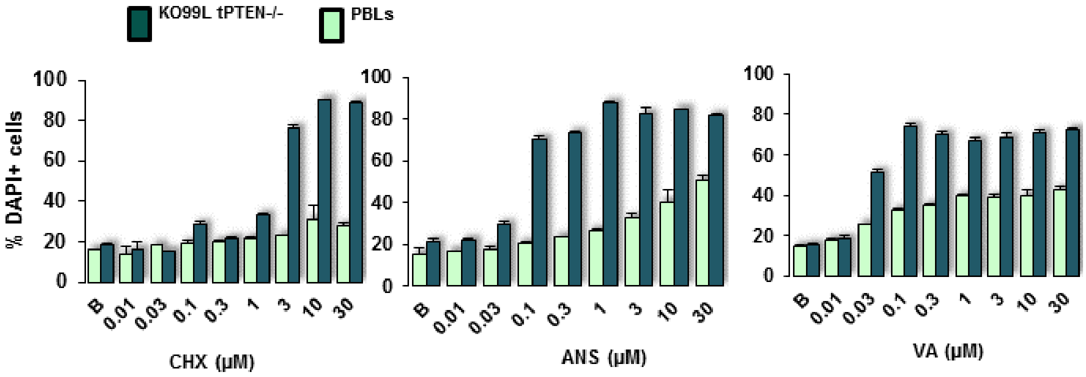

Differential cell death induction by E-specific antibiotics on leukemic versus normal cells

Flow cytometry analysis of cell death induction by the E-specific antibiotics CHX, anisomycin and verrucarin-A on KO99L tPTEN-/- leukemic cells and normal human PBLs, after a 48h incubation period.

Supplementary Fig. 5

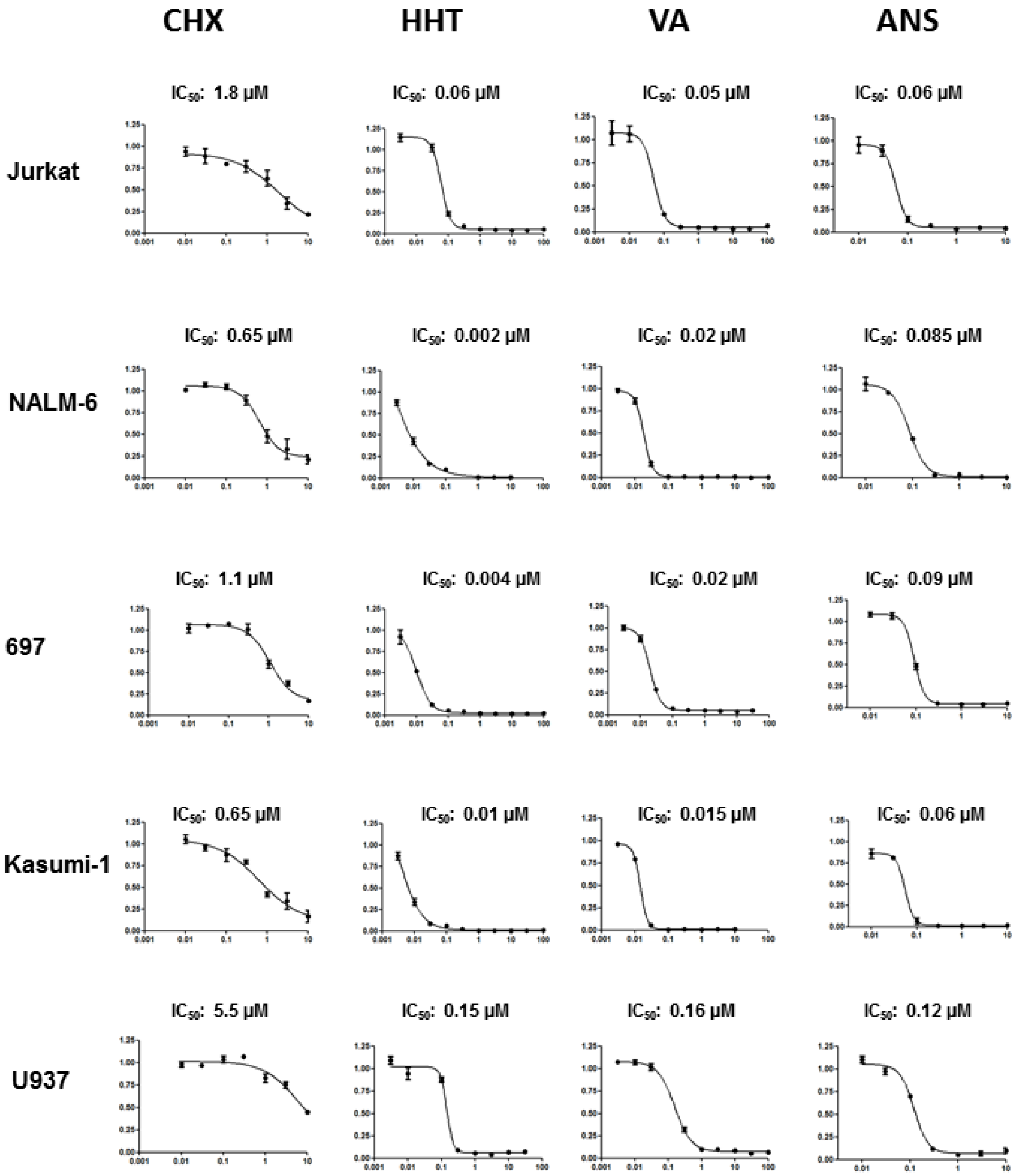

Effect of E-specific antibiotics on leukemic metabolism and survival

Individual curves and IC50 corresponding to Fig. 2G. WST1 assay of mitochondrial activity and cell survival, 48h after incubation of KO99L tPTEN-/- cells with increasing doses of the different compounds; *x* and *y* axis respectively represent drug concentrations in µM and metabolic index (WST1 assay).

Supplementary Fig. 6

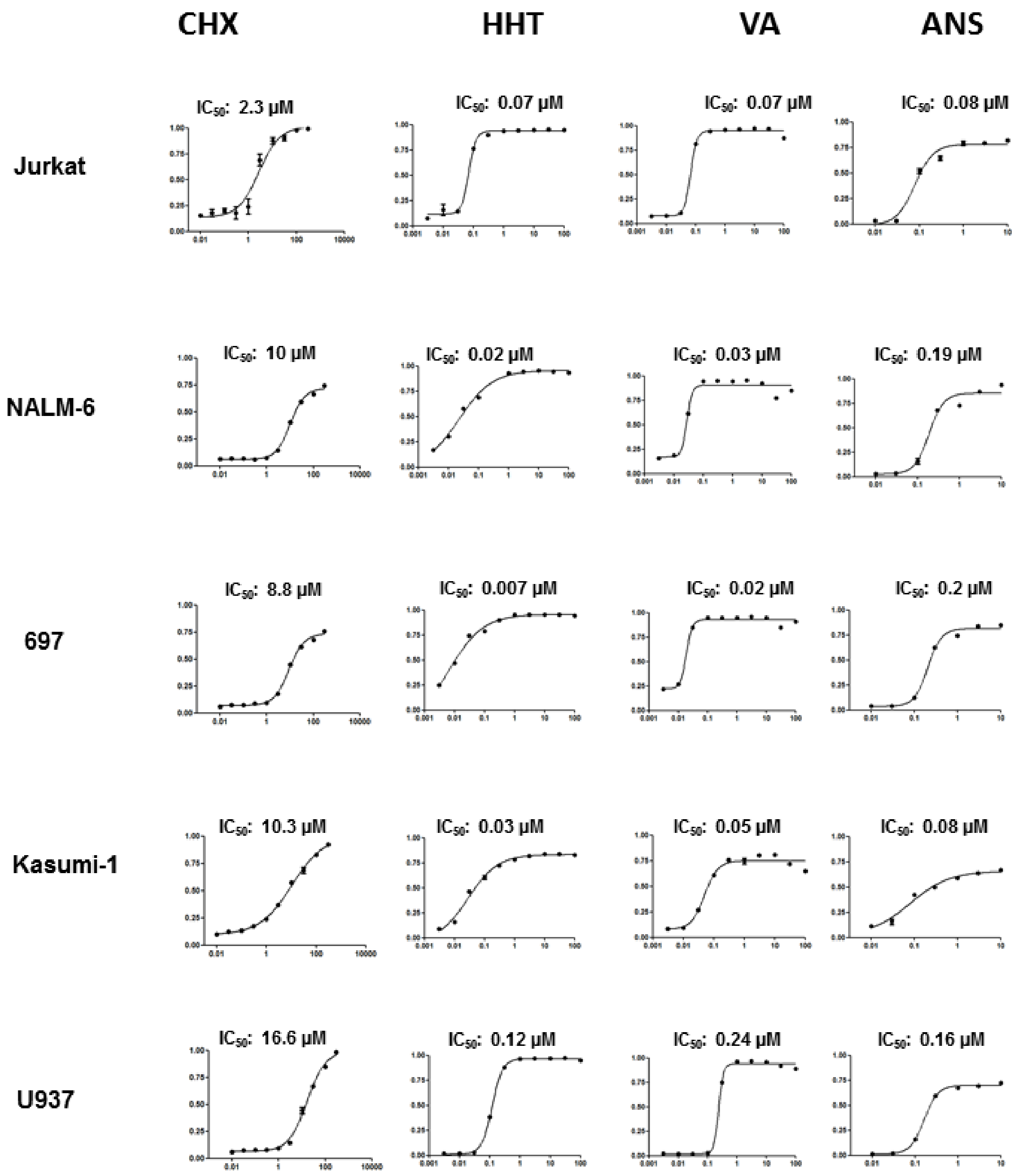

Effect of E-specific antibiotics on leukemic cell death

Individual curves and IC50 corresponding to main **Fig. 2C**. Flow cytometry analysis of cell death induction 48h after incubation of KO99L tPTEN-/- cells with increasing doses of the different compounds; *x* and *y* axis respectively represent drug concentrations in µM and cell death index of DAPI+ cells.

Supplementary Fig. 7

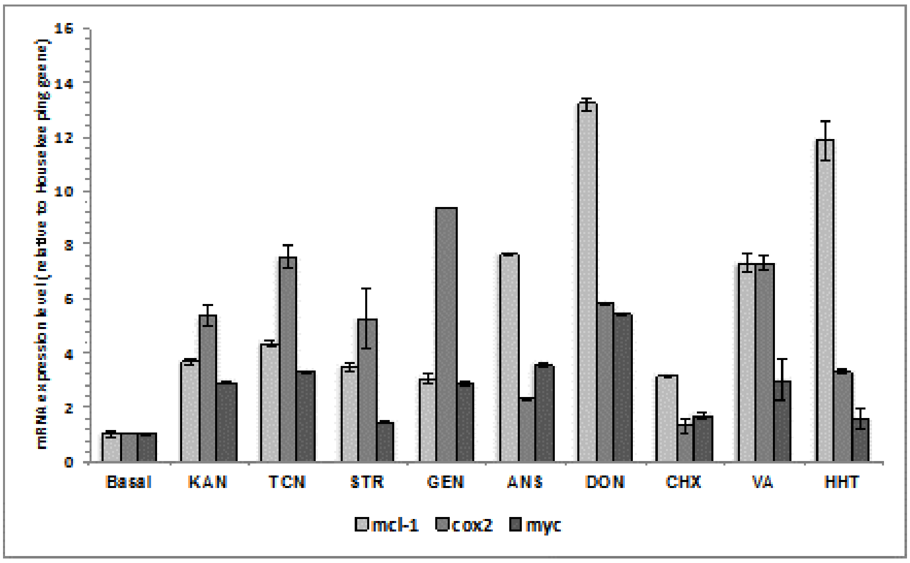

Effect of antibiotics on *c-myc*, *mcl-1* and *cox-2 mRNA levels*

Real-time quantitative PCR of mRNA levels for *c-myc*, *mcl-1* and *cox-2*, 24h after incubation of tPTEN-/- KO99L cells with kanamycin, gentamycin, streptomycin (300.0 µM); tetracyclin (100.0 µM); anisomycin (1.0 µM); CHX (10.0 µM); deoxynivalenol (0.3µM); verrucarin A (0.3 µM); homoharringtonin (0.05 µg/ml) for 24h.

Supplementary Fig. 8
